# Rapid, Nitric Oxide Synthesis-Dependent Activation of MMP-9 at Pericyte Somata During Capillary Ischemia *in vivo*

**DOI:** 10.3389/fphys.2020.619230

**Published:** 2021-01-11

**Authors:** Robert G. Underly, Andy Y. Shih

**Affiliations:** ^1^Department of Neuroscience, Medical University of South Carolina, Charleston, SC, United States; ^2^Center for Developmental Biology and Regenerative Medicine, Seattle Children’s Research Institute, Seattle, WA, United States; ^3^Department of Pediatrics, University of Washington, Seattle, WA, United States; ^4^Department of Bioengineering, University of Washington, Seattle, WA, United States

**Keywords:** pericyte, blood-brain barrier, ischemia, matrix-metalloproteinase-9, capillary, two-photon imaging, nitric oxide, stroke

## Abstract

Nitric oxide serves essential roles in normal vascular physiology, but paradoxically contributes to vascular pathology in disease. During brain ischemia, aberrant nitric oxide levels can cause cellular injury through induction of nitrosative/oxidative stress and post-translational activation of matrix-metalloproteinase-9 (MMP-9). We recently demonstrated that brain pericyte somata were associated with very early and localized MMP-9 activation along capillaries during cerebral ischemia, leading to focal blood-brain barrier disruption. Here, we tested whether this effect was dependent upon nitric oxide production. *In vivo* two-photon imaging was used to directly visualize MMP9 activity using a FITC-gelatin probe and leakage of intravenous dye during photothrombotically induced capillary ischemia. Results showed that the NOS inhibitor, L-NIL, at concentrations affecting both iNOS and constitutive NOS isoforms, attenuated capillary leakage at pericyte soma-specific locations and substantially reduced FITC-gelatin cleavage. We also found that combined administration of L-NIL and anisomycin, an inhibitor of protein synthesis, led to near complete elimination of FITC-gelatin cleavage and vascular leakage. These results indicate that both nitric oxide synthase and new protein synthesis are involved in the rapid activation of MMP-9 at somata of capillary pericytes during ischemia.

## Introduction

During stroke, rapid activation of the proteolytic enzyme, matrix-metalloproteinase-9 (MMP9) leads to degradation of the neurovascular unit and compromise of the blood-brain barrier (BBB) (Gasche et al., 1999; Planas et al., 2001; Montaner, 2003; Fukuda et al., 2004). This process can occur within minutes to hours, and worsens stroke outcome by allowing brain entry of toxic blood molecules and by shortening the time window for thrombolytic treatment. Delineating the mechanisms of early BBB disruption in stroke may identify potential targets for therapeutic intervention. Pericytes, the mural cells of the brain’s capillary bed, have been identified as cellular targets in treatment of acute stroke (Dalkara and Arsava, 2012; Zheng et al., 2020). Pericytes are uniquely sensitive to oxidative/nitrosative stress (Yemisci et al., 2009), and their death or aberrant contraction causes impaired cerebral perfusion during both ischemia and reperfusion phases of stroke (Hall et al., 2014; Hill et al., 2015). However, pericytes also appear to induce MMP-9 activity during neuroinflammation (Bell et al., 2012; Machida et al., 2015) and ischemia (Underly et al., 2017). Using *in vivo* two-photon imaging techniques, we recently showed that pericytes are spatially associated with rapid MMP-9 activation (within 10 min) during ischemia (Underly et al., 2017). This was an unexpected result considering that most prior studies have reported MMP-9 activation hours to days after the ischemic event (Weekman and Wilcock, 2015). To study rapid MMP-9 activity *in vivo*, we used two-photon microscopy to image mice with labeled pericytes, in combination with a well established probe of MMP-9 activity (FITC-gelatin). This was coupled with a photothrombotic model of capillary occlusion to induce ischemia during imaging, and then rapidly track its pathological consequence. These studies revealed extensive overlap between the somata of capillary pericytes, FITC-gelatin cleavage and leakage of blood plasma. These findings point to pericytes as a trigger for rapid MMP-9 activation, likely through induction of existing pools of MMP-9 or rapid MMP-9 expression. However, the prior studies did not delineate the upstream signals involved in pericyte-associated MMP-9 activation.

Nitric oxide (NO) is a diffusible signaling molecule integral for regulation and maintenance of vascular homeostasis (Bredt et al., 1990; Alderton et al., 2001). Nitric oxide is produced through the oxidation of L-arginine by three isoforms of nitric oxide synthase (NOS) which include neuronal NOS (nNOS), endothelial NOS (eNOS), and inducible NOS (iNOS). Two isoforms, nNOS and eNOS, are considered constitutively active in resting cells and also calcium dependent, while iNOS is activated through stimulation by pro-inflammatory cytokines and is calcium independent. Nitric oxide contributes to rapid post-translational modifications, including S-nitrosylation, tyrosine nitration, and S-glutathionylation, which can result in activation of a wide variety of proteins (Martínez-Ruiz et al., 2011; Bradley and Steinert, 2015). S-nitrosylation is the process by which NO reacts with cysteine thiol residues on proteins to form an S-nitrosylated derivative of the protein. Matrix metalloproteinases (MMPs) are a target for S-nitrosylation during pathological conditions involving rapid increases in NO bioavailability through activation of NOS. S-nitrosylation of MMP-9 has been shown to play a role in neuronal cell death (Gu et al., 2002), and may therefore also be involved in the rapid induction of MMP-9 by pericytes during microvascular ischemia. The purpose of this study was to determine if NOS inhibition is sufficient to reduce MMP-9 activation and vascular leakage associated with pericytes during capillary ischemia *in vivo*.

## Results

### Photothrombotically Induced Capillary Ischemia and FITC-Gelatin Imaging *in vivo*

**FIGURE 1:**
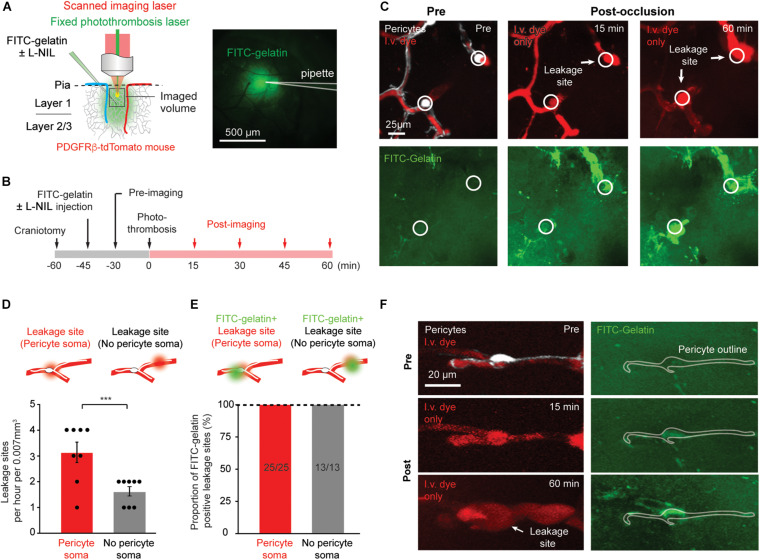
Rapid MMP-9 activation and localized capillary leakage occurs preferentially at pericyte somata. **(A)** Left, Schematic of experimental approach. *In vivo* two-photon imaging is used to visualize multiple structures/processes: PDFGRβ-tdTomato mice for visualization of pericytes, intravenous Texas red-dextran dye for capillary structure and BBB leakage, FITC-gelatin for MMP-9 activity. A Ti:Sapphire scanned laser was used for imaging, while a fixed green continuous wave laser was used for photothrombotic occlusion of capillaries. Right, area of tissue labeled by intracortical FITC-gelatin pressure injection (with or without L-NIL). **(B)** Experimental timeline for surgical procedures and imaging. **(C)** Example of *in vivo* imaging data, with pericytes and/or i.v. dye visible in upper row, and FITC-gelatin fluorescence increase due to its cleavage by active MMP-2/9 in the bottom row. The locations of pericyte somata are marked with a circle. **(D)** Number of focal BBB leakage events occurring at pericyte soma or in non-soma regions. Leakage sites per hour per 0.007 mm^3^: 3.125 ± 0.398 (pericyte soma), 1.625 ± 0.183 (no pericyte soma). Paired *t*-test: *t*(7) = 5.612, ***p = 0.0008, *N* = 8 mice (one region imaged per mouse) for each treatment group. Data is presented as mean ± S.E.M. **(E)** Proportion of leakage sites that are positive for FITC-gelatin cleavage. Each bar shows FITC-gelatin positive leakage sites over total number of leakage sites observed. **(F)** Example of marked co-localization between pericyte soma and early FITC-gelatin activation.

We previously established a method to induce capillary ischemia *in vivo* during two-photon imaging, by using focal photothrombotic approach to occlude small regions of capillary bed in the cerebral cortex (Figure 1A; Underly and Shih, 2017). In this model, capillary ischemia results in cessation of flow in a population of capillaries (∼10–12 capillary segments) that are collectively contacted by ∼3–5 capillary pericytes. These ischemic capillary segments experience rapid peri-vascular activation of MMP-9, followed by blood plasma leakage. The sites of MMP-9 activation and leakage appear focal, and occur with twofold to threefold greater likelihood at capillary regions occupied by pericyte somata (Underly et al., 2017). Pericyte soma location was ascertained by using a mouse line in which pericytes were fluorescently labeled (PDGFRβ-tdTomato). These mice were intravenously injected with a 70 kDa Texas red-dextran dye to visualize the microvasculature (Figures 1B–D). The mice were further intracortically injected with a small volume of MMP-2/9 sensitive probe, FITC-gelatin, at the time of cranial window implantation. Altogether, this preparation enabled concurrent imaging of capillary pericyte location, MMP-2/9 activity, microvascular structure, and BBB breakdown with high spatiotemporal resolution *in vivo*. In conjunction with the photothrombotic model, which allows imaging immediately post-occlusion, very early pathological events associated with ischemia could be observed.

### Plasma Leakage Occurs Predominately at Pericyte Somata While FITC-Gelatin Cleavage Occurs at All Leakage Sites

We first sought to reproduce results observed previously (Underly et al., 2017). We tracked the regions of capillary ischemia before photothrombosis, and every 15 min after photo-thrombosis for a period of 60 min (Figure 1B). As ischemia progressed, heterogeneous plasma leakage was observed, which manifested as bright fluorescent foci at the site of leakage onset, surrounded by a diffuse border of fluorescence. Since multiple time-points were imaged, we could identify the original location of leakage, even if the leaked dye diffused away in later time-points. Leakage sites occurred only following complete cessation of flow in the associated capillary segment (Figure 1C). We counted the number of leakage sites at locations containing pericyte somata and those devoid of pericyte somata. Consistent with our prior study, leakage sites occurred with significantly greater frequency (twofold) at pericyte somata compared to regions lacking somata (Figure 1D). All leakage sites contained localized increases in FITC-gelatin fluorescence, irrespective of pericyte location (Figure 1E). At very early time-points following photothrombosis, FITC-gelatin fluorescence could be seen prior to vascular leakage (Figure 1E, FITC-gelatin, 2nd row). In many cases, clear spatial overlap could be seen between this early FITC-gelatin accumulation and the tdTomato-positive pericyte soma, suggesting that the pericytes themselves might be a source of active MMP-9 (Figure 1F). Critically, our past work also showed that FITC-gelatin cleavage at pericyte somata was dependent on MMP-9, rather than MMP-2 using isoform specific inhibitors (Underly et al., 2017). We therefore refer to FITC-gelatin cleavage also as MMP9 activation in this context.

### Pericyte Leakage Is Attenuated With a Concentration of L-NIL That Inhibits eNOS/nNOS, but Not iNOS Alone

**FIGURE 2:**
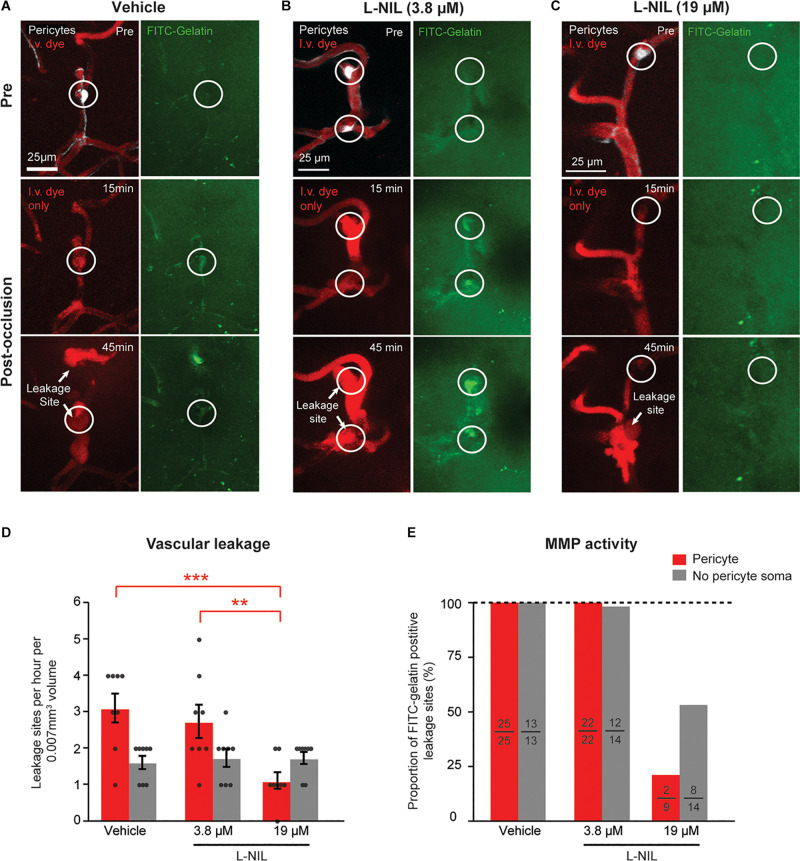
Dose-dependent reduction of pericyte-associated BBB leakage and MMP-9 activity with intracortical L-NIL injection. **(A)** Representative example of vehicle injected control mouse (same example as in panel of Figure 1F). **(B,C)** Representative example of mice receiving low-dose (3.8 μM) and high-dose (19 μM) L-NIL. **(D)** Effect of L-NIL on number of BBB leakage occurring at pericyte soma and non-soma locations. Pericyte soma leakage sites per hour per 0.007 mm^3^: 3.125 ± 0.398 (vehicle), 2.750 ± 0.453 (3.8 μM L-NIL), 1.125 ± 0.227 (19 μM L-NIL); No pericyte soma leakage sites per hour per 0.007 mm^3^: 1.625 ± 0.183 (vehicle), 1.750 ± 0.250 (3.8 μM L-NIL), 1.750 ± 0.164 (19 μM L-NIL). One-way ANOVA with Bonferroni *post hoc* test; *F*(5, 42) = 6.373, *p = 0.045 overall, ***p = 0.0004 (vehicle soma vs. 19 μM L-NIL soma), **p = 0.0052 (3.8 μM L-NIL soma vs. 19 μM L-NIL soma); *N* = 8 mice (one region imaged per mouse) for each treatment group. Data is presented as mean ± S.E.M. **(E)** Proportion of leakage sites with FITC-gelatin cleavage at pericyte soma and non-soma locations with and without L-NIL. Each bar shows FITC-gelatin positive leakage sites over total number of leakage sites observed.

To determine if NOS activity could be involved in the observed rapid activation of MMP-9, we intra-cortically injected the moderately selective iNOS inhibitor, L-N6-(1-Iminoethyl)lysine (L-NIL), together with FITC-gelatin 30 min prior to photothrombosis. Two doses of L-NIL were tested: A lower dose expected to inhibit primarily iNOS (3.8 μM) and a higher dose (19 μM) expected to begin inhibiting constitutive forms, nNOS and eNOS as well. The low-dose of 3.8 μM affected neither the occurrence of vascular leakage nor MMP-9 activation (Figures 2A,B,D,E). However, the higher dose (19 μM) significantly reduced leakage events and MMP-9 activation occurring at pericyte soma locations (Figures 2C–E). High-dose L-NIL had no effect on vascular leakage at locations lacking pericyte somata. FITC-gelatin cleavage was also reduced at leakage sites lacking pericyte somata, albeit to a lesser extent than at pericyte soma locations. Collectively, these data suggests that constitutive NOS forms (probably eNOS, for which L-NIL is slightly more selective) may be contributing to the overproduction of NO and rapid activation of MMP-9 at pericyte somata during capillary ischemia.

### NOS Activity and Protein Synthesis Additively Inhibit Vascular Leakage and FITC-Gelatin Cleavage

**FIGURE 3:**
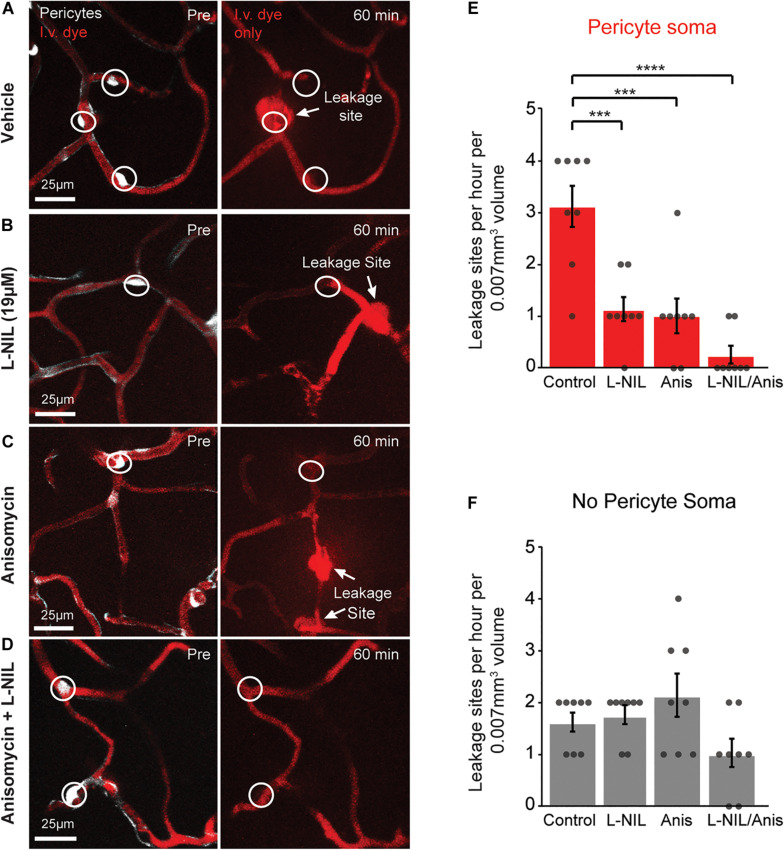
Additive effect of L-NIL and anisomycin on pericyte-associated BBB leakage. **(A–D)** Representative examples of pericyte-associated BBB leakage in the vehicle group **(A)**, and with administration of high-dose L-NIL **(B)**, anisomycin **(C)**, and L-NIL + anisomycin **(D)**. **(E)** Effect of inhibitors on incidence of BBB leakage at pericyte somata. Pericyte soma leakage sites per hour per 0.007 mm^3^: 3.125 ± 0.398 (veh), 1.125 ± 0.227 (19 μM L-NIL), 1.000 ± 0.327 (anisomycin), 0.250 ± 0.164 (19 μM L-NIL + anisomycin). One-way ANOVA with Bonferroni *post hoc* test; *F*(3, 28) = 17.58, ***p < 0.0001 overall, ***p = 0.0003 (vehicle vs. 19 μM L-NIL), ***p = 0.001 (vehicle vs. anisomycin), ****p < 0.0001 (vehicle vs. L-NIL + anisomycin); *N* = 8 mice (one region imaged per mouse) for each treatment group. Data is presented as mean ± S.E.M. **(F)** Effect of inhibitors on incidence of BBB leakage at non-soma locations. Non-soma leakage sites per hour per 0.007 mm^3^: 1.625 ± 0.183 (veh), 1.750 ± 0.164 (19 μM L-NIL), 2.125 ± 0.398 (anisomycin), 1.000 ± 0.267 (19 μM L-NIL + anisomycin). One-way ANOVA with Bonferroni *post hoc* test; *F*(3, 28) = 3.015, *p* = 0.05 overall, non-significant; *N* = 8 mice (one region imaged per mouse) for each treatment group. Data is presented as mean ± SEM.

We further asked whether rapid MMP-9 activation was entirely a post-translational process or also involved new protein synthesis. To test this, we administered the protein synthesis inhibitor, anisomycin (75 mg/kg, i.p.), either alone or in combination with intra-cortical injection of 19 μM L-NIL (Figure 3). Anisomycin is known the pass the BBB following i.p. injection, and has been shown to inhibit brain protein synthesis by ∼90% within 10 min after peripheral injection (Flood et al., 1973). Anisomycin reduced the number of leakage sites at pericyte somata significantly (Figure 3E), but had no effect on leakage sites away from pericyte somata (Figure 3F). The combination of high-dose L-NIL and anisomycin produced an additive reduction in leakage sites at pericyte somata, nearly eliminating their occurrence entirely (Figures 3E,F). The combination treatment had no effect on leakage sites lacking pericyte somata (Figure 3F).

**FIGURE 4:**
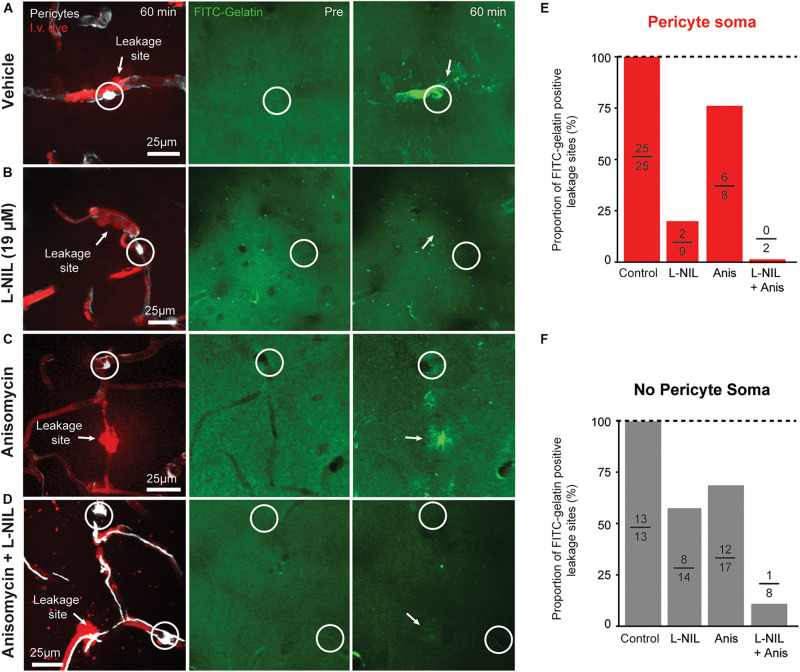
Additive effect of L-NIL and anisomycin on MMP-9 activity at both pericyte soma and non-soma locations. **(A–D)** Representative examples of pericyte-associated FITC-gelatin activation in the vehicle group **(A)**, and with administration of high-dose L-NIL **(B)**, anisomycin **(C)**, and L-NIL + anisomycin **(D)**. **(E,F)** Effect of inhibitors on proportion of leakage sites with FITC-gelatin activity at pericyte soma or non-soma locations. Each bar shows FITC-gelatin positive leakage sites over total number of leakage sites observed.

We further compared the effects of anisomycin, alone or in combination with L-NIL on MMP-9 activity at leakage locations (Figure 4). Anisomycin reduced the percentage of FITC-gelatin positive leakage sites at pericyte somata by 25% (Figure 4E) and the percentage of non-pericyte leakage sites by 29% (Figure 4F). When the inhibitors were administered together, pericyte-specific leakage sites that were FITC-gelatin positive were completely eliminated (Figure 4E) and FITC-gelatin positive non-pericyte leakage sites were reduced by 87% (Figure 4F). Collectively, these results suggest that post-translation modification of MMPs, via NO, contributes more than protein synthesis in the activation of MMP 2/9 during capillary ischemia. However, both post-translational modification and new expression of MMP-9 may be involved in rapid activation of MMP-9 activity.

## Discussion

We have previously shown that pericytes are involved in very early BBB damage during ischemia, and that the vascular damage near their somata corresponds to rapid increases MMP-9 activity (Underly et al., 2017). The speed of this activation implied a post-translational modification of existing pro-MMP-9 pools. We extend these prior studies by examining NO production as a potential upstream signal that leads to this MMP-9 activation. Specifically, we show that pharmacological inhibition of NOS with L-NIL significantly reduces the number of sites of BBB breakdown. A low-dose that inhibits iNOS selectively had no effect, while higher dose that begins to inhibit both nNOS and eNOS as well, blocked both leakage and MMP-9 activity. We also find that MMP-9 activation can be further ameliorated with an inhibitor of protein synthesis, although NO-dependent mechanisms seemed to be the predominant pathway. The combination of both L-NIL and anisomycin led to an additive effect and led to near complete blockade of MMP-9 activity and leakage at pericyte somata, suggesting both mechanisms are at play. From this evidence we speculate that a post-translational modification of MMP-9 by NO (Gu et al., 2002) may be one of the key mechanisms of pericyte-associated BBB breakdown during ischemia. It also suggests that some of the salutary actions of NO inhibition in experimental stroke studies may, in part, be through reduction of pericyte-associated MMP9 activation and BBB disruption (Willmot et al., 2005).

Our findings add further weight to the notion of pericyte sensitivity in vascular pathology by showing that NO can act on pericyte MMP-9 activation very rapidly after ischemic onset. This is complementary to prior work showing that pericytes are exquisitely sensitive to nitrosative/oxidative stress during brain ischemia and (Yemisci et al., 2009). Yemisci et al. (2009) used NO scavengers to reduce aberrant pericyte contraction that could lead to impaired microcirculatory perfusion. More recently, it was shown that amyloid beta induces oxidase stress in pericytes in part through activation of NADPH oxidase 4 (Nox4) in pericytes, leading to their aberrant contraction (Nortley et al., 2019). In the context of amyloid beta toxicity, the NOS blocker L-NNA did not alter pericyte contraction, suggesting against a role for reactive nitrogen species in pericyte contraction and death. However, this may be different in the more rapid and severe pathology of ischemia, where reactive nitrogen species are likely to be produced. Indeed, a recent study by Nishimura et al. (2016) showed marked increase of Nox4 expression in brain pericytes after middle cerebral artery occlusion stroke, which corresponded with BBB breakdown. In parallel studies using pericyte culture, they linked Nox4 over-expression to upregulation of MMP9 gene expression through increased phosphorylation of NFκB. These gene expression changes were seen over longer durations of time after stroke (24–48 h) than the very acute stages studied here. However, they delineate a potential pathway through which anisomycin could block rapid, protein synthesis-dependent MMP9 activity in pericytes during ischemia.

While the source of NO is not entirely clear, it could conceivably be produced by the endothelium, which expresses RNA for eNOS (Nos1) at high levels, but also iNOS (Nos2) at lower levels in the normal brain microvasculature (Vanlandewijck et al., 2018). The proximate localization of the endothelium and pericytes makes for effective NO-MMP-9 interaction. Over-abundance of NO could also be derived from locally affected neurons that express nNOS. Though less well understood, mitochondrial NOS (mtNOS) may also be a potential source of NO from pericytes. Curiously, pericytes are more densely packed with mitochondria, compared to other neurovascular cell types, suggesting that mtNOS may be more prevalent in pericytes (Mathiisen et al., 2010). We further speculate that pericytes are a source of MMP-9 that can be rapidly expressed, or deployed in existing pro-enzyme pools. This is as opposed to infiltration of neutrophils, which have been described as a major source of MMP-9 in cerebral ischemia (reviewed by Turner and Sharp, 2016). Evidence for this comes from observation of clear co-localization in shape between tdTomato-positive pericytes and early FITC-gelatin activation. Also, the initiation of MMP-9 activity occurs in capillaries lacking blood flow, which could not support the recruitment of neutrophils. Other studies have also suggested that pericytes are a source of MMP-9 *in vivo* (Bell et al., 2012). However, further genetic studies to delete MMP-9 specifically in pericytes at adulthood will be required to fully assess this possibility. Additionally, future work is also necessary to prove that the direct S-nitrosylation of MMP-9 occurs in pericytes. A further limitation is that anisomycin does not solely block the synthesis of MMP9. It is possible that inhibition of other MMP9 regulating proteins was involved in the observed reduction of MMP9 activity.

In summary, our results provide a basis for deeper mechanistic studies on pericytes as inducers of BBB disruption in stroke, and potentially other cerebrovascular diseases were MMP-9 has been implicated in microvascular pathology (Weekman and Wilcock, 2015). Pericytes are in direct apposition to the capillary endothelium making them a particularly ill-placed source of unchecked proteolytic activity. If they are indeed the source of rapid MMP-9 activation during ischemia, their pathological contributions to BBB damage may be significant, widespread, and readily overlooked by other biochemical approaches lacking spatiotemporal resolution to resolve the process. While aberrant NO and MMP-9 are well-established moderators of BBB damage during acute ischemia, our work has added pericytes as a site of convergence for these mechanisms.

## Methods

### Mice

Tdtomato reporter mice (Ai14) on a C57/Bl6 background were purchased from Jackson Labs (Jax #: 007914) (Madisen et al., 2010). These mice were crossed with PDGFRβ-Cre mice (Cuttler et al., 2011) to achieve transgene expression in pericytes. Mice were maintained in standard cages on a 12 h light-dark cycle, and housed five or less per cage. Both male and female mice were used, and all mice used were between 2 and 5 months of age.

### Surgery

Skull-removed, dura-intact craniotomies (Mostany and Portera-Cailliau, 2008) were generated over the sensorimotor cortex to achieve optical access for two-photon imaging. Intracortical injections of FITC-gelatin and other drugs were performed (see below) when the craniotomies were generated and animals were imaged immediately after sealing the craniotomy with a coverslip. Anesthesia was induced with isoflurane (Patterson Veterinary) at 3% mean alveolar concentration in 100% oxygen and maintained at 1–2% during surgery. Body temperature was maintained at 37°C with a feedback-regulated heat pad. All animals were administered buprenorphine for analgesia prior to surgery at a concentration of 0.025 mg/kg.

### *In vivo* Two-Photon Microscopy

Two-photon imaging was performed with a Sutter Moveable Objective Microscope and a Coherent Ultra II Ti:Sapphire laser source. A wavelength of 800 nm was used for excitation of FITC-gelatin and Texas red-dextran. Excitation was tuned to 975 nm to successively capture tdTomato fluorescence at the start of each experiment. Green and red emission was simultaneously collected using ET525/70m and ET605/70m filter sets, respectively (Chroma Corp.). Throughout the duration of imaging, mice were maintained under light isoflurane (0.75%) supplied in medical air (20–22% oxygen and 78% nitrogen, moisturized by bubbling through water; AirGas Inc.). Pulse oximetry (MouseOx; Starr Life Sciences) was used to monitor blood oxygen saturation and heart rate to ensure that cardiovascular function was normal during imaging.

Procedures for vascular imaging and analysis have been described previously (Shih et al., 2012). Briefly, the blood plasma was labeled by infraorbital vein injection of 0.025 mL of Texas red-dextran (70 kDa, D-1830; Life Technologies) prepared at a concentration of 5% (w/v) in sterile saline. A 4-X, 0.13 NA air objective lens (UPLFLN 4X; Olympus) was used to generate vascular maps of the entire window for navigational purposes. High-resolution imaging was performed using a water immersion 20-X, 1.0 NA objective lens (XLUMPLFLN 20XW; Olympus). Image stacks for data analysis consisted of 200 μm (x) × 200 μm (y) × 150 μm (z) volumes of cortex (sensory region) starting from the pial surface. FITC-gelatin was homogeneously seen throughout this volume of tissue.

### Photothrombotic Occlusions

Restricted photothrombotic capillary occlusions have been previous described (Underly and Shih, 2017). Briefly, a focused green laser (1 mW, 20 μm diameter at focal plane) was applied directly to the superficial capillary bed (avoiding pericyte somata) for 25 s following an infraorbital injection of Rose Bengal (0.013 mg/kg). This led to the diffuse irradiation of a population of capillaries surrounding the focal point of the laser.

### Quantification of Capillary Leakage Sites

Image stacks collected *in vivo* using two-photon microscopy were rendered in 3-D using Imaris 7.7 Software (Bitplane). Lateral sampling was 0.41 μm per pixel and axial sampling was 1 μm per pixel. Vascular leakage was quantified from 3-D renderings of the Texas red-dextran labeled channel, and also confirmed in 2-dimensional images by scrolling through z-stacks with Fiji/ImageJ software. This method of quantification was previous described (Underly et al., 2017; Underly and Shih, 2017). Briefly, leakage events were defined as the localized permeation of Texas red-dextran from the intravascular space into the surrounding parenchyma. The sites of leakage were separated into pericyte soma-specific and non-pericyte soma leakage sites based on the localization of the pericyte soma to the central portion of the intravascular dye extravasation. These leakage sites were counted at each time point to give a cumulative total of the number of sites during the 1 h duration of the experiment.

### *In vivo* Gelatin Zymography

A FITC-gelatin probe (Bozdagi et al., 2007) (DQ-Gelatin, D12054; Life Technologies), diluted to a concentration of 1 mg/mL in sterile PBS, was pressure injected into the cortex using a pulled glass pipette (10–20 μm tip diameter). The pipette tip was lowered 250 μm into the cortex following the removal of a circular portion of skull (∼2 mm) over the somatosensory area. A small incision was made in the dura mater using a 26-gauge syringe needle tip to facilitate entry of the glass pipette. FITC-gelatin was injected over 5 min using a Picospritzer (10–20 ms pulses, 5–15 psi, 0.5–2 Hz pulse frequency) until 200 nL was delivered. The injection pipette was then left in place for 10 min before removal from the cortex. The craniotomy was then overlaid with 1.5% agarose, as described previously (Shih et al., 2012), followed by a circular coverslip. The coverslip was fixed in position with dental cement prior to two-photon imaging. Quantification of “MMP-positive” cells was previously described (Underly et al., 2017). Cells that co-localized with pericyte somata (tdTomato) were counted as FITC-gelatin positive pericytes. Areas of heightened FITC-fluorescence that did not co-localize with pericyte somata, but exhibited some cellular morphology, were counted as FITC-gelatin positive non-pericytes.

### Nitric Oxide Synthase Inhibition

L-NIL was prepared into stock solutions by dissolving 1 mg/mL L-NIL in sterile PBS and then diluting to working concentrations (3.8, 19 μM) in PBS, together with the FITC-gelatin, which was then injected intracortically.

### Protein Synthesis Inhibition

Anisomycin was administered intraperitoneally at a concentration of 75 mg per kg of mouse body weight. Anisomycin was prepared by adding the minimum amount of 1 M HCL required to bring the drug into solution. Sodium hydroxide (1 M) was then added to bring the solution to a neutral pH. Saline was then added to bring the solution to the appropriate concentration (Wanisch and Wotjak, 2008). Intraperitoneal administration of anisomycin was done 30 min prior to taking the pre-occlusion imaging stack for the experiments. Capillary occlusions were made ∼35 min following anisomycin administration.

### Statistics

All statistical analyses were performing using Graphpad PRISM 9 software. Statistical details are provided in the legend of each figure.

## Data Availability Statement

The raw data supporting the conclusions of this article will be made available by the authors, without undue reservation, to any qualified researcher. Source data is provided in Supplementary Material.

## Ethics Statement

The animal study was reviewed and approved by IACUC of the Medical University of South Carolina.

## Author Contributions

RU performed the data collection and analysis. RU and AS wrote the manuscript. Both authors contributed to the article and approved the submitted version.

## Conflict of Interest

The authors declare that the research was conducted in the absence of any commercial or financial relationships that could be construed as a potential conflict of interest.

## Abbreviations

BBB: blood-brain barrier
MMP-9: matrix metalloproteinase-9
NOS: nitric oxide synthase
iNOS: inducible nitric oxide synthase
eNOS: endothelial nitric oxide synthase
nNOS: neuronal nitric oxide synthase
PDGFR β: platelet-derived growth factor beta
L-NIL: L-N 6-(1-Iminoethyl)lysine.
